# Birth weight as a predictor of breast cancer: a case–control study in Norway

**DOI:** 10.1038/sj.bjc.6600011

**Published:** 2002-01-07

**Authors:** L J Vatten, B O Mæhle, T I Lund Nilsen, S Tretli, C-c Hsieh, D Trichopoulos, S O Stuver

**Affiliations:** Department of Community Medicine and General Practice, The Norwegian University of Science and Technology, N-7489 Trondheim, Norway; Department of Pathology, University of Bergen, Norway; The Norwegian Cancer Registry, Oslo, Norway; Department of Epidemiology and Center for Cancer Prevention, Harvard School of Public Health, Boston, Massachusetts, USA; University of Massachusetts Cancer Center, Worcester, Massachusetts, USA

**Keywords:** breast cancer, birth weight, birth length, placenta weight

## Abstract

The hypothesis that birth weight is positively associated with adult risk of breast cancer implies that factors related to intrauterine growth may be important for the development of this malignancy. Using stored birth records from the two main hospitals in Trondheim and Bergen, Norway, we collected information on birth weight, birth length and placenta weight among 373 women who developed breast cancer. From the same archives, we selected as controls 1150 women of identical age as the cases without a history of breast cancer. Information on age at first birth and parity were collected from the Central Person Registry in Norway. Based on conditional logistic regression analysis, breast cancer risk was positively associated with birth weight and with birth length (*P* for trend=0.02). Birth weights in the highest quartile (3730 g or more) were associated with 40% higher risk (odds ratio, 1.4, 95% confidence interval, 1.1–1.9) of breast cancer compared to birth weights in the lowest quartile (less than 3090 g). For birth length, the odds ratio for women who were 51.5 cm or more (highest quartile) was 1.3 (95% confidence interval, 1.0–1.8) compared to being less than 50 cm (lowest quartile) at birth. Adjustment for age at first birth and parity did not change these estimates. Placenta weight was not associated with breast cancer risk. This study provides strong evidence that intrauterine factors may influence future risk of breast cancer. A common feature of such factors would be their ability to stimulate foetal growth and, simultaneously, to influence intrauterine development of the mammary gland.

*British Journal of Cancer* (2002) **86**, 89–91. DOI: 10.1038/sj/bjc/6600011 www.bjcancer.com

© 2002 The Cancer Research Campaign

## 

The hypothesis that breast cancer may originate *in utero* implies that factors related to intrauterine growth in the female offspring increase adult risk of breast cancer (Trichopoulos, 1990). It has been suggested that prenatal stimulation of growth may increase the number of cells and the rate of cell division in breast tissue and, thereby, increase the risk of malignant transformation (Russo *et al*, 1982; Anbazhagan and Gusterson, 1994). Studies assessing birth weight as a predictor of breast cancer have, however, reached different conclusions. The first large study was conducted in Sweden, using birth records, and showed a modest positive association between birth weight and breast cancer risk (Ekbom *et al*, 1992). In a second study that included birth record information from four additional hospitals, the same researchers could not confirm this result (Ekbom *et al*, 1997). Two other case–control studies in the US also showed no clear relation of birth weight with breast cancer (Le Marchand *et al*, 1988; Sanderson *et al* ((1998))), although in one study a positive association for premenopausal women was suggested (Sanderson *et al* ((1998)). In contrast, a nested case–control study within the Nurses Health Study cohort found a positive association between birth weight and breast cancer risk (Michels *et al*, 1996). Most recently, one cohort study reported a weak positive association (Stavola *et al*, 2000), and the results of three case–control studies also showed positive but weak associations (Innes *et al*, 2000; Hübinette *et al*, 2001; Kajser *et al*, 2001).

In a review of the hypothesis that intrauterine exposures may affect adult breast cancer risk, Potischman and Troisi (1999) concluded that the question remains unresolved and that more studies are warranted. Since historical records of perinatal information are available in Scandinavian countries (Ekbom *et al*, 1992, 1997), we conducted a case–control study of breast cancer using birth records of two large hospitals in Norway. Our primary objective was to assess the association between birth size (i.e., weight and length) and breast cancer risk based on direct measurements obtained at birth. Using available information we could also adjust for potential confounding by the established risk factors of age at first birth and parity, which were not available in the Swedish data (Ekbom *et al*, 1992, 1997).

## MATERIALS AND METHODS

The population base of this study comprised all female residents of Trondheim and Bergen, Norway, who were born at the two main hospitals in those cities between 1910 and 1970. In order to identify eligible participants, we first used the Norwegian Cancer Registry to identify 1035 breast cancer cases who were residents in these cities at the time of diagnosis. The Central Person Registry, a department of Statistics Norway, provided the necessary information to ascertain that 719 of these cases were also born in Trondheim or Bergen. This registry maintains continuously updated records on each woman's residential and childbearing history, including her mother's identity. For each case of breast cancer, we used the Central Person Registry to select four women (*n*=2876) with no history of breast cancer. These potential controls were born consecutively to the case in the same city and were residents of Trondheim or Bergen at the time of the case's diagnosis.

In order to identify which cases and controls were born at the two main obstetric hospital departments in Trondheim and Bergen, we used the identity of the mother to locate the correct birth record files, since the mother was the index person in the birth record archives. For the 719 potential cases, birth records were identified for 187 in Trondheim, and 186 in Bergen. In Trondheim, we aimed at identifying four controls per case, and were able to find 669 (3.6 per case). Due to limited resources, we aimed at identifying three controls per case in Bergen, and found 481 controls (2.6 per case). Thus, birth records were identified for 1150 of the 2876 potential control women who were born at the two main hospitals (Table 1

**Table 1:**
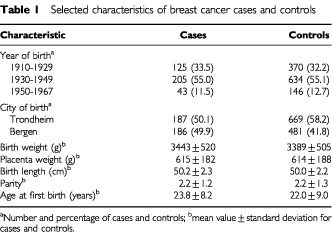
Selected characteristics of breast cancer cases and controls

). The 373 cases were diagnosed between 1959 and 1997, with a mean age at diagnosis of 50 years (range 27–83 years). The other women who were initially identified as potentially eligible for the study were either born at home or at smaller hospitals that are no longer in operation. Information from these sources has not been systematically stored, and was not used in the study.

From the available birth records, we abstracted perinatal information on birth weight (grams), birth length (centimetres), and placenta weight (grams). We also collected maternal information about height, marital status, and father's occupation; which was available for nearly half of the participants. The information on childbearing history (i.e., age at first birth, parity) of the cases and controls was collected from the Central Person Registry in Norway. We used conditional logistic regression to examine the effect of birth weight, length, and placenta weight on breast cancer risk, categorizing each study factor by quartiles, based on the distribution among controls, and making adjustment for age at first birth and parity.

## RESULTS

As expected, increasing age at first birth was associated with increasing risk of breast cancer, and there was a reduction in risk with increasing parity (Table 2

**Table 2:**
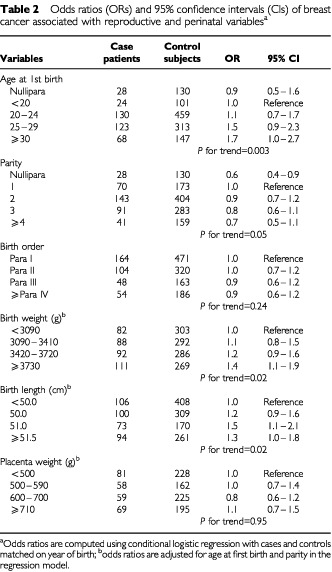
Odds ratios (ORs) and 95% confidence intervals (CIs) of breast cancer associated with reproductive and perinatal variables^a^

), and for birth order, there was no association. We found a positive association between birth weight and breast cancer risk (Table 2, *P* for trend=0.02). Birth weight in the highest quartile (⩾3730 g) was associated with an odds ratio of 1.4 (95% confidence interval=1.1–1.9) compared to the lowest quartile (⩾3090 g). For birth length, there was a similar positive association (*P* for trend=0.02), with an odds ratio of 1.3 (95% confidence interval=1.0–1.8), for the highest (⩾51.5 cm) versus the lowest quartile (<50 cm). Adjustment for indicators of the mother's socio-economic status (i.e., maternal height, marital status, father's occupation) did not materially change these estimates in the subset of subjects for whom this information was available (data not shown). There was no association between placenta weight and breast cancer risk (Table 2).

## DISCUSSION

Studies of the effect of birth weight on breast cancer risk have yielded different results (Le Marchand *et al*, 1988; Ekbom *et al*, 1992, 1997; Michels *et al*, 1996; Sanderson *et al*, (1998); Innes *et al*, 2000; Stavola *et al*, 2000; Hübinette *et al*, 2001; Kajser *et al*, 2001). The initial Swedish studies (Ekbom *et al*, 1992, 1997) employed a study design that was similar to that of the present study. However, a weakness of these studies was the lack of information on age at first birth and parity. Although information on these factors was available in the prospective Nurses Health Study (Michels *et al*, 1996) and could be adjusted for in the statistical analysis, recorded information on birth weight could not be retrieved from birth records. Instead, the mothers of the nurses provided this information, either from memory or from personal files. Major strengths of the present study were the availability of accurate measurements of birth size contained in the birth records as well as the ability to adjust for potential confounding by age at first birth and parity.

Birth length is typically measured by the half centimetre, resulting in a narrow variation compared to that of birth weight, which is measured in grams. Nonetheless, we found a positive association between birth length and breast cancer risk, and the strength of the association was fairly similar to the one related to birth weight. Previous studies with information on birth length have found only weak, and not statistically significant associations between birth length and breast cancer risk (Ekbom *et al*, 1992, 1997; Hübinette *et al*, 2001).

We found no evidence for any association between placenta weight and breast cancer. Previously, placenta weight has only been examined in the Swedish studies (Ekbom *et al*, 1992, 1997) that also found no association with breast cancer risk. In a separate study, however, the same research group reported a positive association between placenta weight and high risk mammographic patterns (Ekbom *et al*, 1995). There is a positive correlation between birth weight and placenta weight (Heinonen *et al*, 2001), and it is conceivable that an effect of birth weight on breast cancer risk could be mediated through pregnancy factors produced by the placenta.

The positive association observed between birth size and breast cancer in the present study could be confounded by socio-economic factors. We did have some information that could indicate socio-economic differences, such as father's occupation, the mother's marital status at birth, and mother's height. The latter could also be correlated with the offspring's birth size for genetic reasons. Due to missing data on these factors for a substantial proportion of the participants we could not resolve this issue. Nonetheless, we did explore the potential confounding by these factors among participants for whom this information was available, but the main results were not substantially altered.

This study provides strong evidence that intrauterine factors may influence future risk of breast cancer. Since positive associations were found for birth weight and birth length, the relevant underlying factors would most likely be linked to fetal growth. A common feature of such factors would therefore be their ability to stimulate fetal growth and, simultaneously, to influence intrauterine development of the mammary gland.
